# Extracellular synthesis of CeNPs and ZnNPs using thermophilic *Anoxybacillus flavithermus and Geobacillus stearothermophilus* bacteria, and their biological activities

**DOI:** 10.1007/s13205-026-04907-8

**Published:** 2026-07-09

**Authors:** Zelal Isik, Sadin Özdemir, Aysin Guzel Deger, Serpil Kizildamar, Erkan Yılmaz, Mustafa Soylak, Nadir Dizge

**Affiliations:** 1 Department of Pharmaceutical Microbiology, Faculty of Pharmacy, Mersin University, 33343 Yenisehir, Mersin Turkey; 2 Department of Food Processing, Technical Sciences Vocational School, Mersin University, 33343 Yenisehir, Mersin Turkey; 3 Department of Analytical Chemistry, Faculty of Pharmacy, Erciyes University, Kayseri, Turkey; 4 Nanotechnology Application and Research Center, ERNAM Erciyes University, 38039 Kayseri, Turkey; 5 Technology Research and Application Center (TAUM), Erciyes University, 38039 Kayseri, Turkey; 6 Department of Chemistry, Faculty of Sciences, Erciyes University, Kayseri, Turkey

**Keywords:** Nanoparticles, Antioxidant, Antimicrobial activity, Microbial cell viability, Biofilm inhibition, Seedling growth

## Abstract

Nanoparticles (NPs) synthesized via biological routes have gained significant attention due to their eco-friendly production and diverse biological applications. However, relatively few studies have explored the use of thermophilic bacteria for nanoparticle synthesis, despite their potential advantages in stability and enzyme robustness under extreme conditions. In particular, thermophilic microorganisms remain under explored for the extracellular biosynthesis of metal nanoparticles such as cerium (Ce) and zinc (Zn), and their comparative biological activities have not been sufficiently investigated. This study aimed to synthesize Ce and Zn nanoparticles using thermophilic bacterial strains and to evaluate their antimicrobial, antioxidant, DNA interaction, antibiofilm, microbial cytotoxic, and plant growth-promoting activities. Ce and Zn nanoparticles were synthesized extracellularly using *Anoxybacillus flavithermus* strain Gecek19 (T19-CeNPs and T19-ZnNPs) and *Geobacillus stearothermophilus* strain Gecek20 (T20-CeNPs and T20-ZnNPs). The biological activities of the nanoparticles were evaluated at different concentrations. Antioxidant activity was assessed using DPPH and metal chelating assays (12.5–200 mg/L). DNA cleavage, microbial cell viability (125–500 mg/L), biofilm inhibition (125–500 mg/L), antimicrobial activity, and seed germination assays (12.5–500 mg/L) were also performed. DPPH radical scavenging activities were 73.45%, 86.46%, 76.03%, and 82.60% for T19-CeNPs, T19-ZnNPs, T20-CeNPs, and T20-ZnNPs, respectively. Metal chelating activities were 67.12%, 64.56%, 72.40%, and 70.35%, respectively. The highest antimicrobial activity was observed for T19-ZnNPs with a Minimum Inhibitory Concentration (MIC) of 64 mg/L against *Staphylococcus aureus* and *Enterococcus faecalis*. All nanoparticles exhibited single-stranded DNA cleavage activity and significantly reduced microbial cell viability. Biofilm inhibition reached up to 99.14% with T19-ZnNPs against *S. aureus*. Additionally, nanoparticle treatments showed positive effects on barley seed germination, particularly enhancing root and coleoptile development at appropriate concentrations. The study demonstrates that thermophilic bacteria are effective biofactories for Ce and Zn nanoparticle synthesis. The resulting nanoparticles exhibited strong multifunctional biological activities, including antioxidant, antimicrobial, antibiofilm, and plant growth-promoting effects, indicating their potential for biomedical and agricultural applications.

## Introduction

Today, nanotechnology is used in many fields with the rapidly developing and increasing demand in the fields of agriculture, food, medicine, textile, aviation and environment. Nanoparticles are more advantageous than macro-size materials in terms of both physical and chemical properties. These materials, which can be synthesized in nanometer size (1–100 nm), are called nanomaterials (Chausali et al. 2022).

The application potential and economic viability of nanomaterials have become central considerations in translating laboratory-scale synthesis into real-world technologies. Nanomaterials, particularly those produced via green and microbial routes, offer significant advantages such as low-energy synthesis, reduced use of toxic reagents, and scalability, which collectively improve their cost-effectiveness compared to conventional physical and chemical methods. Recent studies have demonstrated that biologically synthesized metal nanoparticles can be effectively applied in antimicrobial coatings, wastewater treatment, agriculture, and biomedical systems due to their multifunctional properties and high surface reactivity (Iravani 2011; Singh et al. 2016). Furthermore, the utilization of thermophilic microorganisms can enhance process stability under harsh conditions, potentially reducing operational costs, and increasing yield consistency (Thakkar et al. 2010). From an economic perspective, integrating waste substrates or low-cost microbial culture media further improves feasibility by minimizing raw material expenses. However, large-scale commercialization still requires optimization of downstream processing, recovery efficiency, and regulatory validation to ensure cost competitiveness and environmental safety (Khan et al. 2019). Overall, while challenges remain, nanomaterials synthesized through sustainable biological approaches present a promising balance between performance, environmental impact, and economic viability.

Nanomaterials are used in different fields of medicine, enabling the production of innovative equipment in the health sector (Ceylan et al. 2020). Especially metal-containing nanomaterials are frequently used in the field of medicine because of their antibacterial properties. The antibacterial effect of heavy metal containing nanomaterials on Gram-positive and Gram-negative bacteria is known (Doğaç et al. 2024). The antibacterial effect is realized by the photocatalytic activity of semiconductor heavy metals, which is their unique feature, or by the production of radicals that occur with the release of metallic ions (Bazin 2022). Yeo et al. (2022) published a review article in which they investigated the synthesis of alternative materials due to the success of antibiotics in inhibiting bacteria, as well as antibiotic resistance in overconsumption, the ability of some bacteria to break the effect of antibiotics and other determined harmful effects. In their studies, they focused on the ability of metal-containing nanomaterials to produce reactive oxygen and to destroy bacteria by destroying the cell wall, DNA and proteins of the bacteria with the release of ions. A review on the use of antibacterial properties of metal-containing nanoparticles in the field of livestock was presented by Michalak et al. (2022). In their studies, they stated that heavy metals can be an alternative to antibiotics and also promote their health. They emphasized that when nanoparticles containing heavy metals such as selenium and zinc are taken by the living thing, harmful bacteria in the intestine can be eliminated.

While ZnO nanoparticles are generally considered relatively biocompatible at low concentrations, several studies have demonstrated that they can induce cytotoxicity, oxidative stress, and organ damage at higher doses. For example, ZnO nanoparticles have been shown to cause oxidative stress, mitochondrial damage, and liver injury in vitro and in vivo, with toxicity strongly dependent on concentration and exposure conditions (Pei et al. 2022). Similarly, dose-dependent hematological and biochemical alterations have been reported in animal studies exposed to ZnO nanoparticles (Srivastav et al. 2016). Nanomaterials containing zinc oxide (ZnO) do not need a light source to show antibacterial activity. In other words, even if it does not fully show its photocatalytic activity, it stops or even destroys the growth of bacteria in good efficiency (Onyszko et al. 2022). Hossain et al. (2022) synthesized the nanomaterial containing ZnO by precipitation method. They examined the antibacterial properties of the synthesized materials against *E. coli*. They observed that the bacterial viability gradually decreased with the increase in the amount of ZnO in the bacterial medium. They stated that when they combined this material with other nanomaterials, the number of bacteria killed at lower concentrations could increase. However, they emphasized that it has similar inhibition with ZnO alone. In addition, the formation of free radicals is triggered during the oxidation of nanoparticles containing Cerium (CeNPs). These radicals induce oxidative stress in the bacteria and can show good antibacterial properties. As a result of this emerging redox activity and regenerative interaction with the surface, it has gained importance in the field of medicine (Liu et al. 2022). Dar et al. (2022) produced Ce-containing nanomaterial as cerium oxide (CeO_2_) by hydrothermal method. The synthesized material was tested against six different bacteria. In their study, they determined that the nanomaterial amount was able to inhibit all bacteria by using 50 µgCeO_2_/mL. The use of biological materials in the synthesis of such nanoparticles positively affects the chemical and physical properties of such materials, and when they are used together with microorganisms, the use of additional chemicals is prevented and supports the formation of their shapes better. For this reason, the use of microorganisms in the synthesis of nanoparticles is carried out either intracellularly or extracellularly (Alfryyan et al. 2022).

Studies in recent years have emphasized in agricultural uses of nanomaterials, especially as plant nutrients and/or supplements. The agricultural applications of nanoparticles obtained by biological methods are increasing day by day. Some engineered nanomaterials like Zn, can show beneficial effects on plants growth (García-López et al. 2018). Singh et al. (2019) reported that moderate concentration (e.g., 62 mg/L) of green ZnONPs show a better increase in root and shoot length in wheat seedlings than other concentration levels or chemically synthesized ZnONPs. Srivastav et al. (2021) showed that low doses of ZnONPs can act as a seed priming agent to achieve better germination and seedling growth, however, they emphasized that the response of plants to ZnONPs is dose-dependent. However, it has been stated in many studies that high doses can have negative and toxic effects on plant growth (Sarkhosh et al. 2022; Sabir et al. 2014). Also, rare element like Ce can act as a bio-stimulatory in various crop plants (Sobarzo-Bernal et al. 2021). Ramírez-Olvera et al. (2018) reported that Ce increased root, shoot length and biomass in the first growth stages of rice seedlings, moreover increased the germination percentage by an average of 36.2%. These results also coincide with findings reported in other studies with cereals (e.g. rice, corn, wheat, barley) (Fashui 2002; He and Loh 2000; Diatloff et al. 2008; Wang et al. 2010). Nevertheless, different studies have reported that high Ce concentration decreased the growth of seedlings, root-shoot length and biomass to varying degrees (Yang et al. 2020; Ramírez-Olvera et al. 2018; Lizzi et al. 2018). NPs with this aspect, it has taken its place in the literature as an open field to be examined.

Most studies have synthesized nanoparticles using the green synthesis method with mesophilic bacteria. However, few studies have been published on thermophilic microorganisms using the green synthesis method. Therefore, in this study, the thermophilic *Anoxybacillus flavithermus* strain Gecek 19 and *Geobacillus stearothermophilus* strain Gecek 20 were used. Zn and Ce nanoparticles were synthesized extracellularly from thermophilic *Anoxybacillus flavithermus* strain Gecek19 (T_19_-CeNPs and T_19_-ZnNPs) and *Geobacillus stearothermophilus* strain Gecek20 (T_20_-CeNPs and T_20_-ZnNPs). These nanoparticles were synthesized and their antibacterial activities, microbial cell viability, DNA fragmentation and biofilm inhibition were investigated in detail. Additionally, the effects of these NPs on seed germination and early seedling growth were studied to demonstrate environmental impacts and plant growth performance.

## Materials and methods

### Materials

Cerium nitrate (Ce(NO_3_)_3_·6H_2_O) and zinc sulfate (ZnSO_4_) were purchased from Sigma Aldrich. All chemicals were of analytical reagent grade.

### T_19_ and T_20_ bacteria isolation procedure

In this study, strains of a thermophilic bacteria were used. T_19_ and T_20_ bacteria were isolated using the procedure described and also identification of T_19_ and T_20_ bacteria were given in the previous study (Giray et al. 2024). Thermophilic bacteria were isolated from the soil samples obtained from the Ömer hot springs in Afyonkarahisar province. Serial dilution technique was used for isolation. The samples were then passaged in Nutrient broth (NB) medium and incubated at 55 °C for 24 h. After two enrichment cycles the cultures were plated on a NB medium for 48 h. Morphologically different colonies were transferred to a new medium. The microbial inoculations were performed on starch-based Nutrient Broth (NB) agar medium and incubated at 55 °C for 48 h for determination of amylase production. After 24 h of incubation, Lugol’s iodine solution was poured onto the starch-based Nutrient Broth (NB) agar medium where bacterial growth was observed and incubated at room temperature for 5 min. Then, colonies with transparent zone on a blue background were defined as α-amylase positive. Later, streaked on a new NB agar plate for further examination.

### Synthesis of T_19_ and T_20_ nanoparticles

**Fig. 1:**
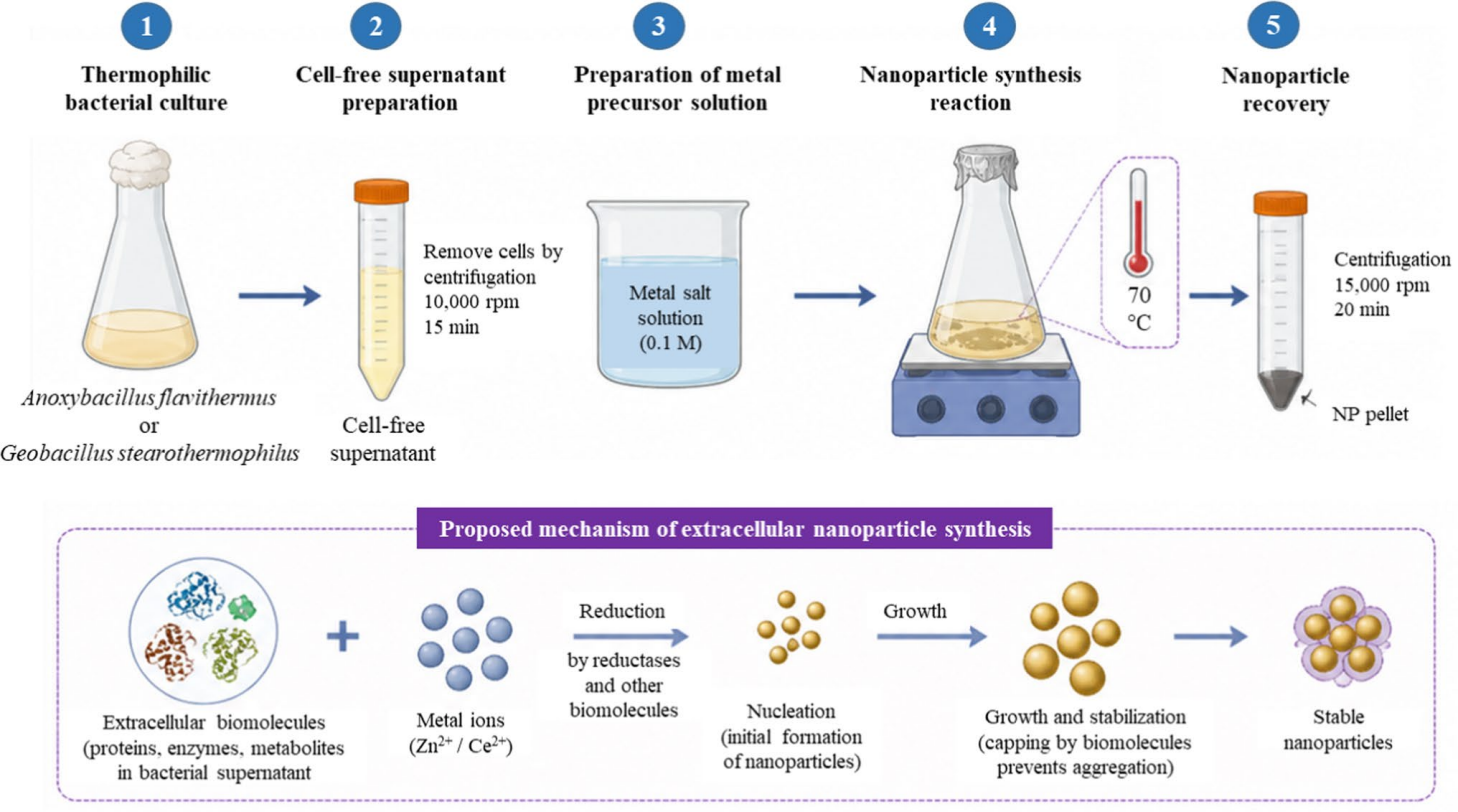
Schematic representation of the experimental setup for the extracellular synthesis of nanoparticles using thermophilic bacterial supernatant

To begin the process of synthesis of Ce and Zn nanoparticles, 0.1 M cerium nitrate and zinc sulfate solutions were prepared. *Anoxybacillus flavithermus* strain Gecek19 (T_19_-CeNPs and T_19_-ZnNPs) and *Geobacillus stearothermophilus* strain Gecek 20 were incubated in NB medium at 55 °C for 48 h in a shaker at 120 rpm by submerged culture. After 48 h of incubation, the microbial culture medium was centrifuged at 10,000 rpm for 15 min. The extracellular culture medium remaining after centrifugation was used directly in the synthesis of nanoparticles. After adding 300 mL of each metal solution to 100 mL of cell free bacterial extracellular medium (containnig 5 g bacteria biomass), the mixture was stirred at 70 °C overnight (Rehman et al. 2019). Since the synthesis was performed using cell-free supernatant, the reaction is governed by extracellular biomolecules rather than active cellular metabolism. Thermophilic bacteria are known to produce thermostable enzymes (e.g., reductases and oxidoreductases) that remain active at elevated temperatures. Operating at 70 °C therefore promotes faster reduction of metal ions, improves reaction kinetics, and facilitates controlled nucleation and growth of nanoparticles, while maintaining enzyme stability. The nanoparticles were washed several times with deionized water and dried for 24 h at 80 °C. Figure 1 show that experimental setup for nanoparticle synthesis. The scheme was prepared as an example for a single bacterial species. The same procedure was applied for both types of bacteria.

### Characterization of T_19_ and T_20_ nanoparticles

The characterization procedures were carried out following standard protocols commonly reported in the literature (Khan et al. 2019; Shaw et al. 2025; Yalcinoz et al. 2026). The synthesized samples were kept in a desiccator under suitable conditions for characterization in order to dehumidify and remain stable. It was used together with emission scanning electron microscopy (FE-SEM; Zeiss/Supra 55) to determine the surface morphologies of the samples and energy dispersive spectroscopy (EDX) to determine the distribution of the elements contained in each sample (Khan et al. 2019). In addition, the crystal structure of the samples was determined according to the results obtained by X-ray diffraction (XRD; Bruker/D8 Venture) (Shaw et al. 2025). The preferred 2θ values for each sample were determined according to previous studies in the literature (Yalcinoz et al. 2026).

### DPPH scavenging activity

All biological activity studies were tested in triplicate. DPPH scavenging activity of T_20_-CeNPs, T_20_-ZnNPs, T_19_-CeNPs and T_19_-ZnNPs was tested with minor modifications of Ağırtaş et al. (2015). A 1000 µL of DPPH methanol solution was incubated for 30 min in the dark with 250 µL of T_20_-CeNPs, T_20_-ZnNPs, T_19_-CeNPs and T_19_-ZnNPs at 12.5, 25, 50, 100, and 200 mg/L. After 30 min, the radical scavenging activity was determined by spectrophotometer at 517 nm. Trolox and Ascorbic acid were taken as standard. The radical scavenging activity was calculated using Eq. (1):

1 $$ Capacity(\% ) = \left( {\frac{{Abs(control) - Abs(sample)}}{{Abs(control)}}} \right) \times 100$$

*Abs*_*control*_ is control absorbance and *Abs*_*sample*_ is the absorbance value of the test compounds and DPPH after 30 min.

### Ferrous chelating activity

The ferrous chelating activity of extracellular green synthesized of T_20_-CeNPs, T_20_-ZnNPs, T_19_-CeNPs and T_19_-ZnNPs was performed by the Dinis process (Dinis et al. 1994). T_20_-CeNPs, T_20_-ZnNPs, T_19_-CeNPs and T_19_-ZnNPs at 12.5, 25, 50, 100, and 200 mg/L were reacted with FeCl_2_ for 2 min and then ferrozine was added. Then, the reaction solution was incubated in the dark for 10 min and the ferrous chelating activity was determined using a spectrophotometer. The absorbance values were measured at 562 nm and the percentage of chelating activity was calculated by Eq. (2):

2 $$Metal\,Chelating\,Effect(\% ) = \left( {\frac{{Abs(control) - Abs(sample)}}{{Abs(control)}}} \right) \times 100$$

Here Abs_control_ represents the absorbance of the control reaction and Abs_sample_ represents the absorbance obtained in the presence of compounds or EDTA.

### DNA cleavage activity

DNA cleavage activity of T_20_-CeNPs, T_20_-ZnNPs, T_19_-CeNPs and T_19_-ZnNPs was determined by agarose gel electrophoresis assay. For this purpose, plasmid pBR322 DNA was used as the target DNA molecule. The plasmid pBR322 DNA used for the study was purchased from Sigma Aldrich. A 5 µL of 0.1 mg/L DNA molecule was exposed to 50, 100, and 200 mg/L concentrations of T_20_-CeNPs, T_20_-ZnNPs, T_19_-CeNPs and T_19_-ZnNPs for 45 min at 37 °C. The loading buffer was added and the plasmid DNA which exposed with test samples were loaded onto a 1.0% agarose gel and stained with EthBr in a Tris-acetic acid-EDTA buffer. Then, agarose gel electrophoresis was performed to visualize the DNA cleavage activity of T_20_-CeNPs, T_20_-ZnNPs, T_19_-CeNPs and T_19_-ZnNPs., the samples were run at 100 V for 1.5 h using an electrophoresis apparatus (Keypour et al. 2015). Plasmid pBR322 DNA were visualized using a transilluminator.

### Antimicrobial activity by microdilution method

Minimum Inhibitory Concentration (MIC) of T_20_-CeNPs, T_20_-ZnNPs, T_19_-CeNPs and T_19_-ZnNPs were evaluated using the 1:1-fold dilution method against *Escherichia coli* (ATCC 10536), *Pseudomonas aeruginosa* (ATCC 9027), *Legionella pneumophila* subsp. *pneumophila* (ATCC 33152), *Enterococcus hirae* (ATCC 10541), *Enterococcus faecalis* (ATCC 29212), *Staphylococcus aureus* (ATCC 6538), *Candida tropicalis* (ATCC 750), and *Candida parapsilosis* (ATCC 22019). Test microorganisms were grown overnight prior to the dilution step. For the study, 1:1-fold serial dilutions of T_20_-CeNPs, T_20_-ZnNPs, T_19_-CeNPs and T_19_-ZnNPs were made and then the above-mentioned microorganisms were added to the microplate wells. Subsequent plates were incubated for 24 h at 37 °C. Antimicrobial activity was evaluated with minimum inhibition concentration (MIC) values defined as the lowest concentration that inhibits microbial growth at the end of 24 h (Giray et al. 2024).

### Biofilm inhibition activity

*S. aureus* (ATCC 6538) and *P. aeruginosa* (ATCC 9027) were used as test microorganisms to determine the effect of T_20_-CeNPs, T_20_-ZnNPs, T_19_-CeNPs and T_19_-ZnNPs synthesized from thermophilic *A. flavithermus* strain Gecek19 and *G. stearothermophilus* strain Gecek 20 on biofilm inhibition. Biofilm inhibition prodedure were done using the procedure described in the previous study (Giray et al. 2024). Well plates containing 125, 250 and 500 mg/L of T_20_-CeNPs, T_20_-ZnNPs, T_19_-CeNPs and T_19_-ZnNPs were inoculated with microorganisms in Nutrient Broth (NB) medium. They were incubated at 37 °C for 72 h. After 72 h, the well plates were emptied slowly and washed twice with distilled water. The plates were dried at 70 °C for 20 min and then 1 mL of 1% crystal violet (CV) was added to stain biofilm for 30 min. The CV was then removed and the plates were gently washed. The washing step was performed twice. Then, 2 mL of 96% ethanol was added and it was waited for 15 min to recover the absorbed CV. Biofilm inhibition was determined by spectrophotometer at 595 nm. Only wells containing *S. aureus* and *P. aeruginosa* were used as positive controls. Biofilm inhibition was calculated according to the Eq. (3).

3 $$Biofilm\,Inhibition(\% ) = \left( {\frac{{Abs(control) - Abs(sample)}}{{Abs(control)}}} \right) \times 100$$

### Microbial cell viability inhibition

*E. coli* (ATCC 10536) was used for cell viability inhibition assay. After the microorganism was inoculated into NB medium, it was incubated in a shaker at 37 °C at 150 rpm for 24 h. After incubation, the fermentation medium was centrifuged at 5000 rpm for 5 min. The bacterial pellet was then washed with sterile saline solution to remove the residue of the fermentation medium. The washed *E. coli* was suspended with NaCl solution. This microbial suspension was used for microbial cell viability inhibition assay. *E. coli* was treated with T_20_-CeNPs, T_20_-ZnNPs, T_19_-CeNPs and T_19_-ZnNPs synthesized from both thermophilic bacteria at 125, 250 and 500 mg/L for 60 min at 37 °C. After 60 min, it was diluted in various ratios and inoculated on NB agar medium and incubated at 37 °C for 24 h. The same application was also studied with the control group that did not contain T_20_-CeNPs, T_20_-ZnNPs, T_19_-CeNPs and T_19_-ZnNPs (Giray et al. 2024). Finally, colonies were counted and microbial cell viability was calculated using Eq. (4).

4 $$\it \% {\text{ Cell viability inhibition}}:\left[ {\left( {{{\mathrm{A}}_{{\mathrm{control}}}}-{{\mathrm{A}}_{{\mathrm{example}}}}} \right)/{{\mathrm{A}}_{{\mathrm{control}}}}} \right] \times 100$$

### Seed germination and root/coleoptile development

Barley “Tarm-92” (*Hordeum vulgare*) seeds were obtained from the Field Crops Central Research Institute of Turkey/Ankara. Seeds were sterilized with a 5% sodium hypochlorite solution for 5 min and rinsed three times with pure water. From 500 mg/L stock nanoparticle suspensions, a total of five dilutions were made with pure water to obtain the following concentrations: 12,5-25-50-100-200 mg/L. Pure water was used as the control group. NP suspensions were freshly prepared by dissolving directly in pure water just before administration and dispersed by ultrasonic vibration (100 W, 40 kHz) for 30 min. In order to avoid nanoparticle aggregation, suspensions were stirred just before use (García-López et al. 2019).Uniform seeds were selected, soaked for 24 h in either aerated pure water or in aerated NPs suspensions containing 12,5-25-50-100-200-500 mg/L. For the germination test, seeds were transferred into 14 cm glass petri dishes containing two-layer Whatman No.1 filter papers, and different concentrations NPs suspensions were added in equal amounts. The same amount of pure water was added to the control groups. A total of 100 seeds, consisting of 4 replicates containing 25 seeds, were used for each application. Germination process was carried out for 3 days at 21 °C in the dark. At the end of the third day, root lengths (RL, mm), coleoptile lengths (CL, mm), germination rates (GR, %), coleoptile rates (CR, %) and seminal root rates (SRR, %) were measured. Seeds were considered germinated when the radicles were ≥ 2 mm long. In addition, seeds were photographed daily for three days.

## Results and discussion

### Characterization of T_19_ and T_20_ nanomaterials

**Fig. 2:**
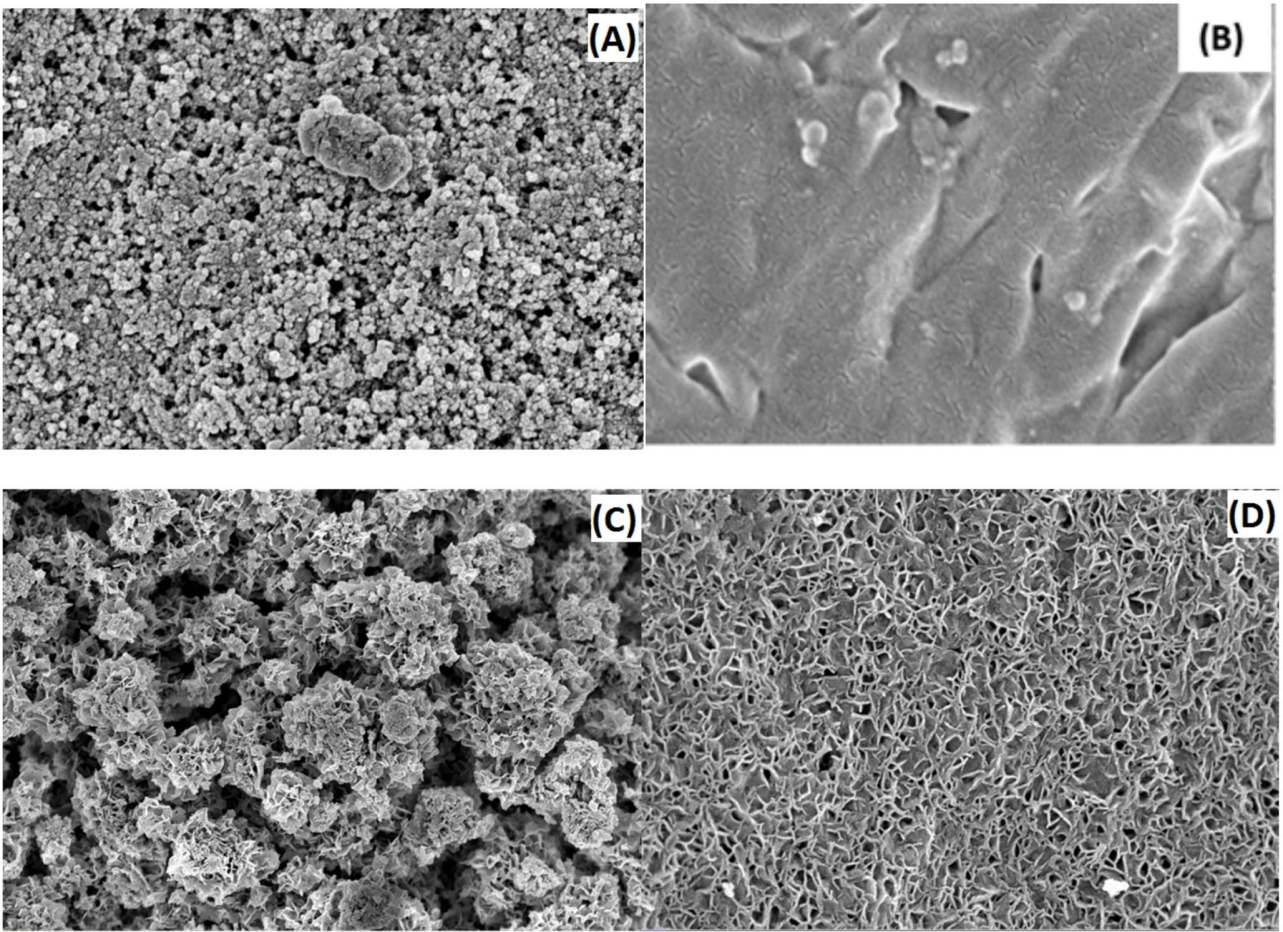
FE-SEM images of nanoparticles, **A** T_19_-CeNPs, **B** T_20_-CeNPs, **C** T_19_-ZnNPs and **D** T_20_-ZnNPs

FE-SEM images of nanoparticles are given in Fig. 2. The surface images of T_19_-CeNPs (200 nm size) show that the material is clustered and there are many pits at the junctions of these clusters. It also showed that T_19_-CeNPs have a particle size of less than 100 nm and were successfully synthesized (Fig. 2A). T_19_-ZnNPs have their own pores on each of them, which are collected in the surface images obtained at 1 μm size. This structure has a partially leafy appearance. Therefore, it has been likened that T_19_-ZnNPs have a flower-like structure (Fig. 2C). In the surface images of T_20_-ZnNPs obtained at 100 nm size, it was observed that there were long and thin structures intertwined with each other. It is present in dispersed spaces between spiral structures. Thus, its surface morphology showed that it had a leaf-like structure (Fig. 2D).

**Fig. 3:**
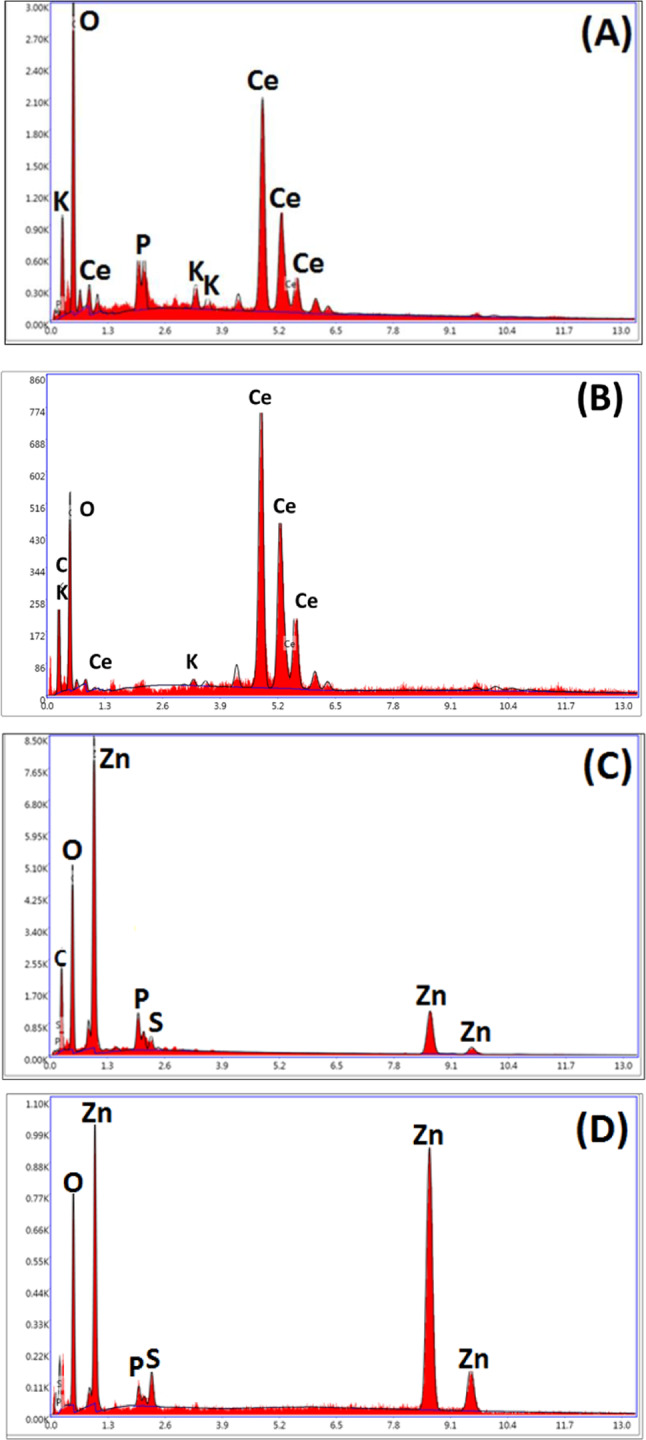
EDX analysis of nanoparticles, **A** T_19_-CeNPs, **B** T_20_-CeNPs, **C** T_19_-ZnNPs and **D** T_20_-ZnNPs

EDX results of nanoparticles synthesized using microorganisms in the study are given in Fig. 3. EDX results of T_19_-CeNPs and T_20_-CeNPs show the presence of high amount of Ce element. This demonstrates its ability to retain Ce in extracellular expression (Fig. 3A and B). Likewise, it can be deduced that T_19_-ZnNPs and T_20_-ZnNPs can be retained by extracellular expression with the presence of high amount of Zn element in EDX results (Fig. 3C and D). Oxygen (O), phosphorus (P) and potassium (K) were detected in the EDX results of all samples belonging to T19 and T20. These elements are thought to come from the microorganisms used during production. The presence of such elements in the cell is necessary for the viability of microorganisms (Rakhmawati et al. 2021).

**Fig. 4:**
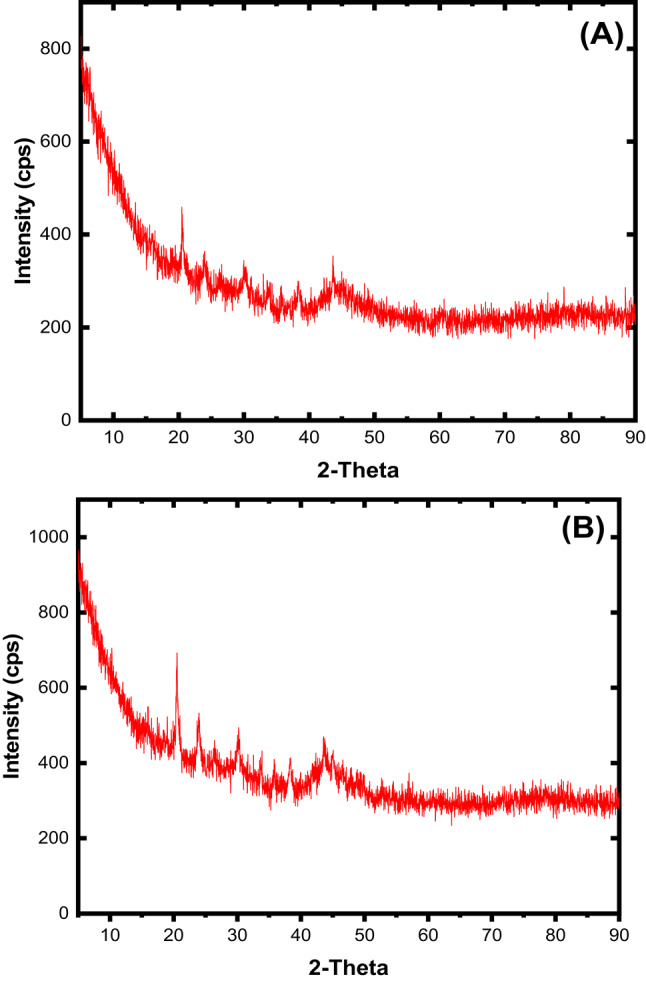

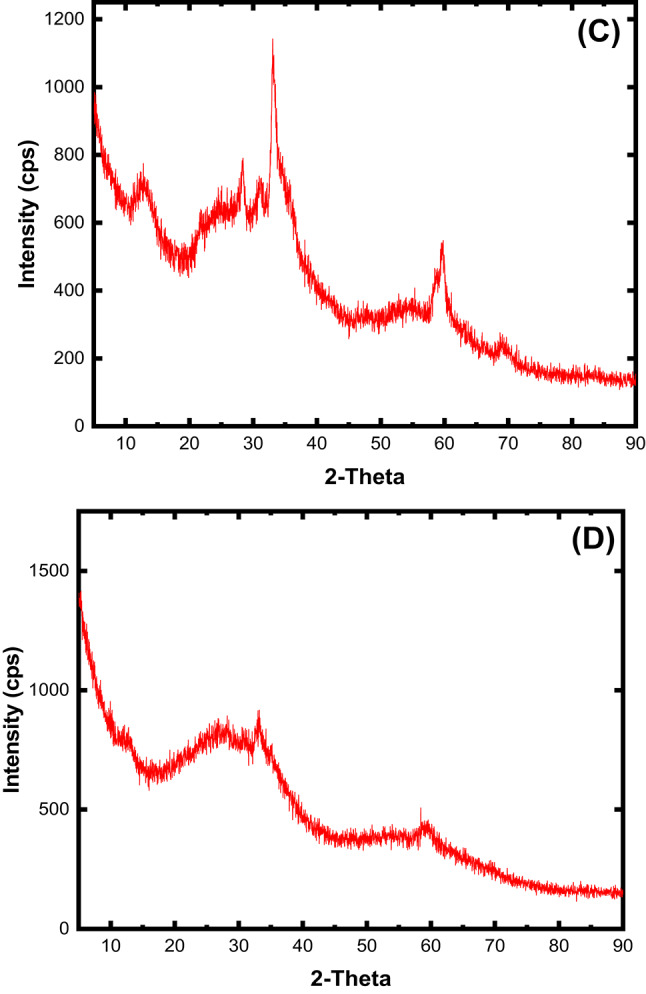
XRD spectrum of nanoparticles, **A** T_19_-CeNPs, **B** T_20_-CeNPs, **C** T_19_-ZnNPs and **D** T_20_-ZnNPs

The crystalline nature of CeNPs and ZnNPs were confirmed by using XRD analysis. XRD analysis were carried out to illuminate crystal structure of T_19_-CeNPs, T_20_-CeNPs, T_19_-ZnNPs NPs, and T_20_-ZnNPs (Fig. 4). When the XRD spectra of T_19_-CeNPs (Fig. 4A) and T_20_-CeNPs (Fig. 4B) were examined, it was observed that almost the same spectra were obtained. The peaks at 2θ = 20.5°, 30.2°, 38.3° and 43.6° proved the formation of Ce_2_O(CO_3_)_2_.2H_2_O NPs. The XRD spectrum of T_19_-ZnNPs NPs (Fig. 4C) and T_20_-ZnNPs (Fig. 4D) was dealt with the standard The International Center for Diffraction Data (ICCD). The XRD spectrum of T_19_-ZnNPs NPs and T_20_-ZnNPs showed diffraction peaks, including 002, 100, 101, 102, 103, 110, 112, 200, and 201 reflection planes of ZnO NPs with hexagonal phase (Doğan and Kocabaş 2020; Kontham et al. 2022; Kurian 2020; Zhang et al. 2021). The all XRD results showed that succcesfull formation of ZnO NPs and Ce_2_O(CO_3_)_2_.2H_2_O NPs were carried by using the developed green synthesis methods.

Although advanced characterization techniques such as FTIR, TEM, DLS, and zeta potential analysis could provide additional insights into surface chemistry, particle size distribution, and colloidal stability, the combination of FE-SEM, EDX, and XRD analyses employed in this study is sufficient to confirm the successful synthesis of ZnO and Ce-based nanoparticles. The XRD results clearly demonstrate the crystalline nature and phase formation, while EDX confirms the elemental composition. FE-SEM images reveal the morphology and aggregation behavior of the nanoparticles. In biologically synthesized nanoparticles, extracellular biomolecules such as proteins and metabolites are known to act as natural capping and stabilizing agents, contributing to particle stability and preventing aggregation. Therefore, the observed structural integrity and reproducible biological activities suggest that the synthesized nanoparticles are sufficiently stable for the investigated applications. Nevertheless, further studies involving detailed surface characterization and long-term stability analysis are recommended for future work.

### DPPH radical scavenging activity

**Fig. 5:**
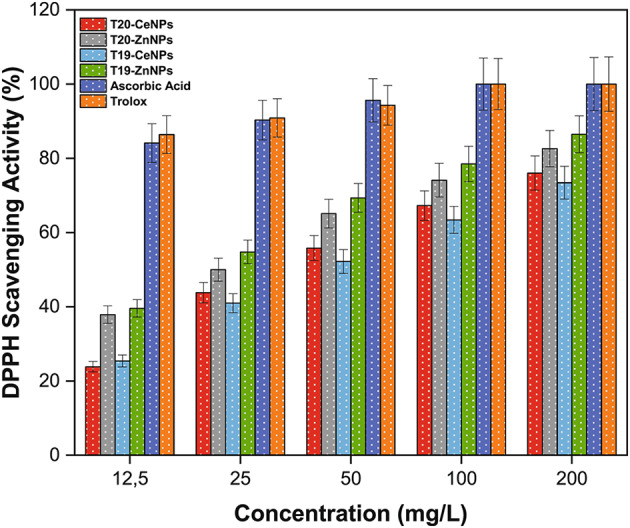
DPPH scavenging activity of T_20_-CeNPs, T_20_-ZnNPs, T_19-_CeNPs, and T_19_-ZnNPs

Antioxidant compounds play a vital role in preventing damage caused by free radicals. Various testing systems are used to discover natural and synthetic compounds with antioxidant effects. One of these test systems is the DPPH radical scavenging method. DPPH, which is a stable radical, is used and antioxidant activity is defined by measuring the ability of the antioxidant to capture this free radical (Pokorny et al. 2001). DPPH assay is the most extensively used assay for determination the antioxidant potential of a substance. In this study, the DPPH scavenging activity of green synthesized T_19_-CeNPs, T_19_-ZnNPs, T_20_-CeNPs, and T_20_-ZnNPs was determined. The results are given Fig. 5. Ascorbic acid and Trolox were used as standards in the study. The DPPH activity of extracellular synthesized Ce and Zn nanoparticles from thermophilic bacteria demonstrated that ZnNPs was better than CeNPs at all concentrations. When we compared the DPPH activities of the nanoparticles according to the bacteria from which they were synthesized, it was found that T_20_-CeNPs > T_19_-CeNPs and T_19_-ZnNPs > T_20_-ZnNPs. When the DPPH activities of T_20_-CeNPs, T_20_-ZnNPs, T_19−_CeNPs, T_19_-ZnNPs increased from 25 mg/L to 100 mg/L, the DPPH activities also increased from 43.81% to 67.26%, from 50.0% to 74.09%, from 40.97% to 63.40%, and from 54.76% to 78.47%, respectively. At 200 mg/L concentrations, DPPH activities were T_19_-ZnNPs (86.46%) > T_20_-ZnNPs (82.60%) > T_20_-CeNPs (76.03%) > T_19_-CeNPs (73.45%). Moreover, according to the results of the study the DPPH activity of the nanoparticles showed a concentration-dependent increase. Gomaji Chaudhary et al. (2020) synthesized cerium oxide nanoparticles using *Cleome simplicifolia* and they informed that its showed concentration-dependent antioxidant activity. Examples of some studies reporting that Ce and Zn nanoparticles have antioxidant activity are as follows. Khalil et al. (2017) reported that the DPPH radical scavenging activity of ZnONPs from *Sageretia thea* (Osbeck) as 63.5% ±2.4% at 200 µg/mL. Sharmila et al. (2019) indicated that *T. castanifolia* leaf extract mediated synthesized ZnO NPs showed 67% DPPH radical scavenging activity at 100 µg/mL. Aseyd Nezhad et al. (2020) found that the radical scavenging activity of synthesized CeONPs originated from *Origanum majorana* were 89.2% at 250 µg/mL concentration. According to our findings, new synthesized CeNPs and especially ZnNPs can be used as antioxidant agents.

### Ferrous chelating activity

**Fig. 6:**
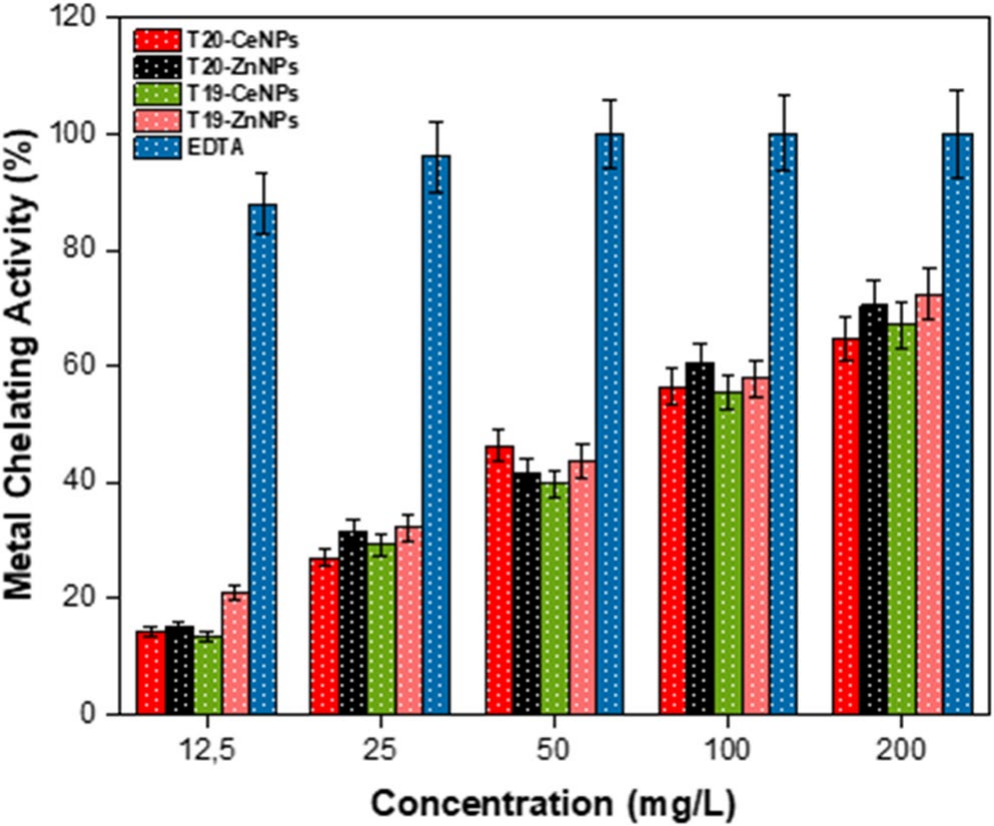
Ferrous chelating activity of T_20_-CeNPs, T_20_-ZnNPs, T_19-_CeNPs, and T_19_-ZnNPs

Reactive oxygen species cause oxidative damage to various biomolecules such as proteins, lipids, and DNA. Iron chelators act as antioxidants by scavenging reactive oxygens (Adjimani and Asare 2015). Metal chelating activity was also studied to determine the antioxidant properties of the synthesized nanoparticles in the study. The results of the ferrous chelating ability of ZnNPs and CeNPs are given in the Fig. 6. In general, according to the results of the ferrous chelating study, it was found that generally ZnNPs had better chelating activity than CeNPs, and also generally T_19_-ZnNPs had better activity than T_20_-ZnNPs. Moreover, as can be observed from the graph, the chelating activities of the nanoparticles were in parallel with the increase in concentration. The chelating activities of EDTA, ZnNPs and CeNPs at 50 mg/L concentration were EDTA (100%) > T_20_-CeNPs (46.33%) > T_19_-ZnNPs (43.61%) > T_20_-ZnNPs (41.73%)> T_19_-CeNPs (39.69%). When the concentration of synthesized nanoparticles increased from 100 mg/L to 200 mg/L, the chelating activities of T_20_-ZnNPs, T_19_-ZnNPs, T_20_-CeNPs and T_19_-CeNPs increased from 60.30% to 70.35%, from 57.75% to 72.40%, from 56.38% to 64.56% and from 55.53% to 67.12% respectively. Balogun and Ashafa (2020) stated that they green synthesized ZnONPs from *Lessertia montana* and it was characterized by several microscopic, spectroscopic and diffraction analysizes. They also investigated the antioxidant activities of green synthesized ZnONPs from *Lessertia montana* and green synthesized ZnONPs exhibited good ferrous chelation activity. Nadaroglu et al. (2017) noticed that they synthesized Ce_2_O_3_NPs by using *Euphorbia amygdaloides* aqueous extract. Green synthesized Ce_2_O_3_NPs was characterized. The chelating Fe^2+^ ability of Ce NPs was investigated and the highest metal chelating activity was found as 71.64%. The results showed that ZnNPs and CeNPs can be used efficiently as metal chelator agent for commercial applications.

### DNA cleavage activity

**Fig. 7:**
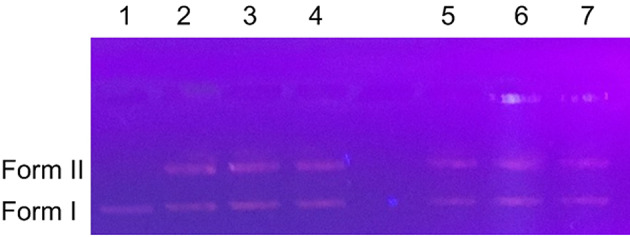
DNA Cleavage activity of T_20_-CeNPs, T_20_-ZnNPs synthesized from thermophilic *A. flavithermus* strain Gecek19. Lane 1, pBR 322 DNA; Lane 2, pBR 322 DNA + 50 mg/L T_20_-CeNPs; Lane 3, pBR 322 DNA + 100 mg/L T_20_-CeNPs; Lane 4, pBR 322 DNA + 200 mg/L T_20_-CeNPs; Lane 5, pBR 322 DNA + 50 mg/L T_20_-ZnNPs; Lane 6, pBR 322 DNA + 100 mg/L T_20_-ZnNPs; Lane 7, pBR 322 DNA + 200 mg/L T_20_-ZnNPs

**Fig. 8:**
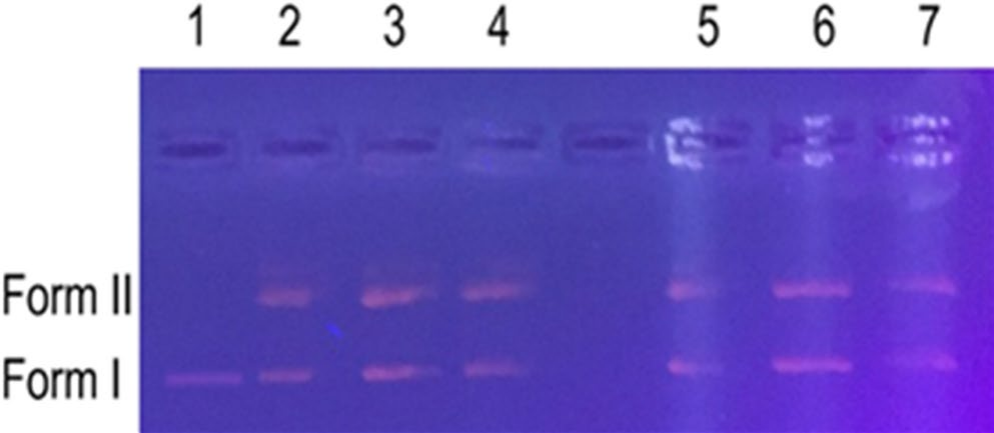
DNA Cleavage activity of T_19_-CeNPs and T_19_-ZnNPs synthesized from *G. stearothermophilus* strain Gecek20. Lane 1, pBR 322 DNA; Lane 2, pBR 322 DNA + 50 mg/L T_19_-CeNPs; Lane 3, pBR 322 DNA + 100 mg/L T_19_-CeNPs; Lane 4, pBR 322 DNA + 200 mg/L T_19_-CeNPs; Lane 5, pBR 322 DNA + 50 mg/L T_19_-ZnNPs; Lane 6, pBR 322 DNA + 100 mg/L T_19_-ZnNPs; Lane 7, pBR 322 DNA + 200 mg/L T_19_-ZnNPs

DNA serves as the central regulatory molecule in cells, governing genetic information storage, transmission, and expression. Thanks to the genetic information it contains, it enables cells to manage vital activities (Pray 2008). Deoxyribonucleic acid (DNA) is the primary target molecule for anticancer and antimicrobial therapy according to cell biology (Raman et al. 2008). In our study, DNA cleavage activity of CeNP and ZnNP synthesized from T18 and T19 bacteria using plasmid pBR322 DNA was investigated. During agarose gel electrophoresis, supercoiled plasmid DNA (Form I) exhibits the highest mobility due to its compact structure. Introduction of a single-strand break converts the supercoiled form into a relaxed open circular structure (Form II), which migrates more slowly through the gel. In contrast, cleavage of both strands produces the linear form (Form III), which displays an intermediate migration pattern between Forms I and II. The DNA cleavage capabilities of T_20_-CeNPs, T_20_-ZnNPs, T_19_-CeNPs and T_19_-ZnNPs are shown in Figs. 7 and 8. Plasmid pBR322 DNA was incubated with 50, 100 and 200 mg/L concentrations of T_20_-CeNPs, T_20_-ZnNPs, T_19_-CeNPs and T_19_-ZnNPs. As seen in Figs. 7 and 8 and T_20_-CeNPs, T_20_-ZnNPs, T_19_-CeNPs and T_19_-ZnNPs synthesized using both thermophilic bacteria induced single strand DNA breakages at all tested concentrations. Guan et al. (2021) produced nanocomposite film (SA-CS@ZnO) consisting of sodium alginate (SA) and chitosan (CS) functionalized with ZnONPs. The effect ZnONPs on the genomic DNA of *E. coli* and *S. aureus* was determined by agarose gel electrophoresis. They suggested that the DNA of *E. coli* and *S. aureus* was damaged and degraded. Ali et al. (2015) prepared and characterized CeONPs. They investigated the CeONPs induce oxidative stress and also they determined the effect of CeONPs on genotoxicity by using human skin melanoma cells. It was detected that a double-strand DNA breakage occured in CeONP-treated cells. These findings showed good similarity with our findings and it can be indicated that T_20_-CeNPs, T_20_-ZnNPs, T_19_-CeNPs and T_19_-ZnNPs can be utilized as agents targeting DNA molecules for various purposes.

### Antimicrobial activity

Micro-dilution process was used to determine the antimicrobial effect of T_20_-CeNPs, T_20_-ZnNPs, T_19_-CeNPs and T_19_-ZnNPs. The Minimum Inhibitory Concentrations (MICs) against test microorganisms were determined to evaluate the antimicrobial efficacy of T_20_-CeNPs, T_20_-ZnNPs, T_19_-CeNPs and T_19_-ZnNPs. The results are shown in Table 1. It was observed that antimicrobial activities differed in each microorganism. MICs of T_20_-ZnNPs were 128 mg/L for *E. coli*, *E. hirae*,* S. aureus* and *C. parapsilosis*; 256 mg/L for *P. aeruginosa*,* L. pneumophila* and *C. tropicalis* and 64 mg/L for *E. fecalis*. MIC values of T_20_-CeNPs were found as 1024 mg/L for *E. coli*, *P. aeruginosa*, and *C. tropicalis*; 512 mg/L for *L. pneumophila*, *E. hirae*,* E. fecalis*,* S. aureus*, and *C. parapsilosis*. MICs of T_19_-ZnNPs extracellularly synthesized from *Anoxybacillus flavithermus* strain Gecek19 were determined as 128 mg/L for *E. coli*, *P. aeruginosa*,* E. hirae*,* C. parapsilosis* and *C. tropicalis*; 256 mg/L for *L. pneumophila*, 64 mg/L for *S. aureus* and *E. fecalis*. MIC values of T_19_-CeNPs were also detected as 1024 mg/L for *E. coli*, *L. pneumophila*, and *C. tropicalis*, 512 mg/L for *C. parapsilosis*, *P. aeruginosa*, *E. hirae*,* E. fecalis* and *S. aureus*. Rehman et al. (2024a) synthesized a nanocomposite using mesoporous metal organic framework (ZIF-8) phyto-fabricated with the herb *Allium sativum* (As). The antimicrobial activity of nanocomposite was assayed against *Candida albicans*, *Shigella flexneri*, and *Candida parapsilosis*. They also reported that nanocomposite demonstrated antimicrobial activity against the *Candida sps*. and bacteria. Irshad et al. (2020) reported that they synthesized ZnONP by green method using *Ocimum basilicum*. They investigated the antimicrobial activity of green synthesized ZnONP and the MIC values of ZnONP obtained from *O*. *basilicum* were found as 312.5 µg/mL for *S. aureus* and *E. coli* as bacterial strains and also 5000 µg/mL for *Aspergillus niger* as fungal strain. The reason for inhibition of bacterial growth by ZnO nanoparticles is that the bacterial cell membrane is damaged, thus resulting in the death of the bacterium (Divyapriya et al. 2014). Rehman et al. (2024b) indicated that they were tested to evaluate antimicrobial ability of silver (Ag)-cisplatin NPs loaded on monodispersed spherical silica against *P. aeruginosa* and *S. aureus*. Their study showed a significant interaction of nanoformulations promoting antimicrobial activities. Khoshgozaran Roudbaneh et al. (2019) investigated the several biological activities of CeONPs and they tested the antimicrobial activity of CeONPs against *S. aureus*, *K. pneumoniae*, *P. aeruginosa* and *E. coli*. They found that CeONPs demonstrated significant antimicrobial activity againts to tested microorganisms especially *E. coli*. They also stated that the reason why CeONPs show higher antibacterial activity against *E. coli* than other bacterial strains. This difference in antimicrobial activity may be due to the different structure and thickness of the cell wall and also particle size, surface charge, and morphology of NPs. According to the antimicrobial activity results especially ZnONPs can be used for antimicrobial applications.

**Table 1:** Antimicrobial activity of T_19_NPs and T_20_NPs

Microorganisms	T_20_-CeNPs	T_20_-ZnNPs	T_19_-CeNPs	T_19_-ZnNPs
*E. coli*	1024	128	1024	128
*P. aeruginosa*	1024	256	512	128
*L. pneumophila*	512	256	1024	256
*E. hirae*	512	128	512	128
*E. fecalis*	512	64	512	64
*S. aureus*	512	128	512	64
*C. parapsilosis*	512	128	512	128
*C. tropicalis*	1024	256	1024	128

### Biofilm inhibition activity

**Fig. 9:**
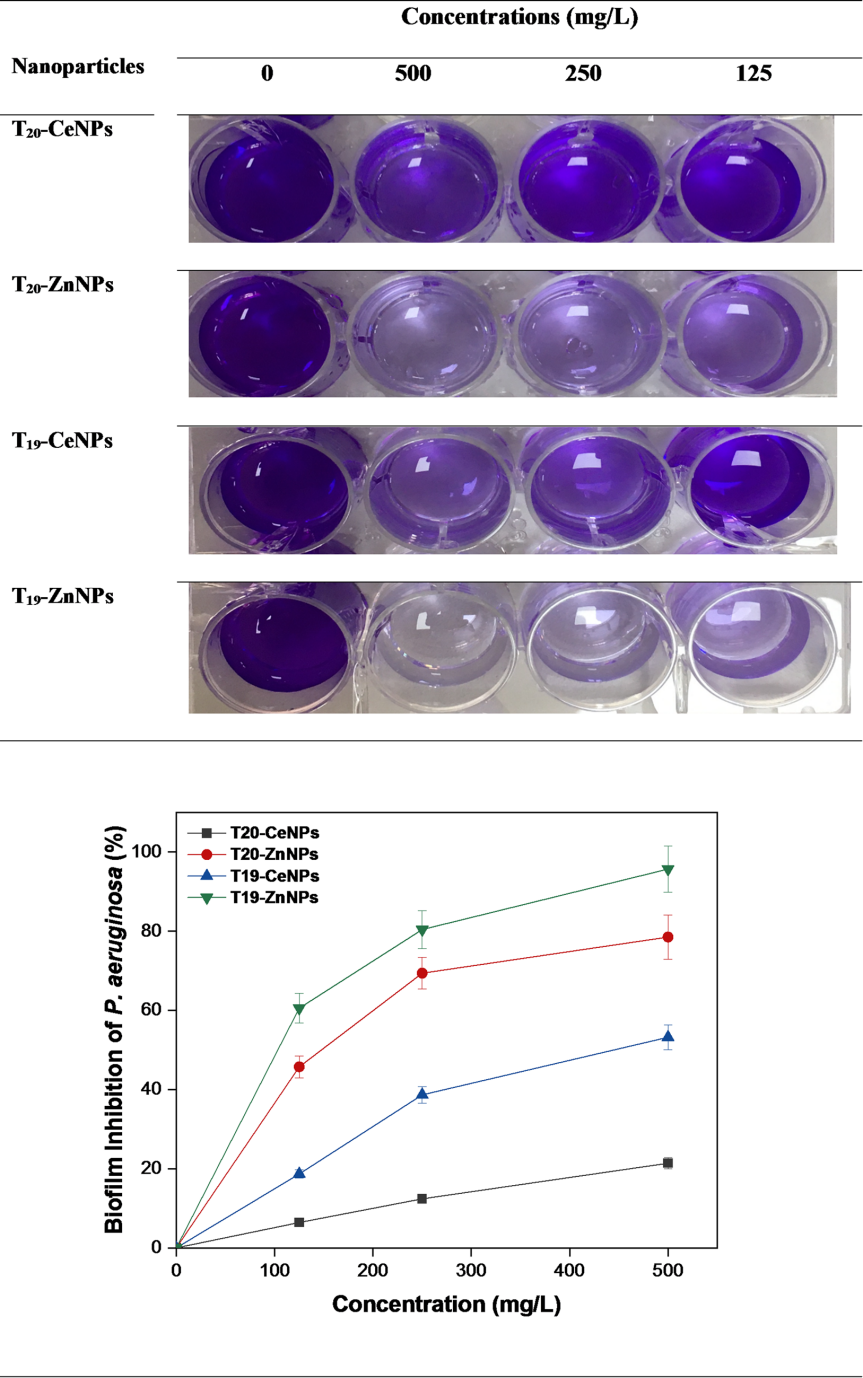
*P. aeruginosa* biofilm inhibition of T_20_-CeNPs, T_20_-ZnNPs, T_19-_CeNPs, and T_19_-ZnNPs

**Fig. 10:**
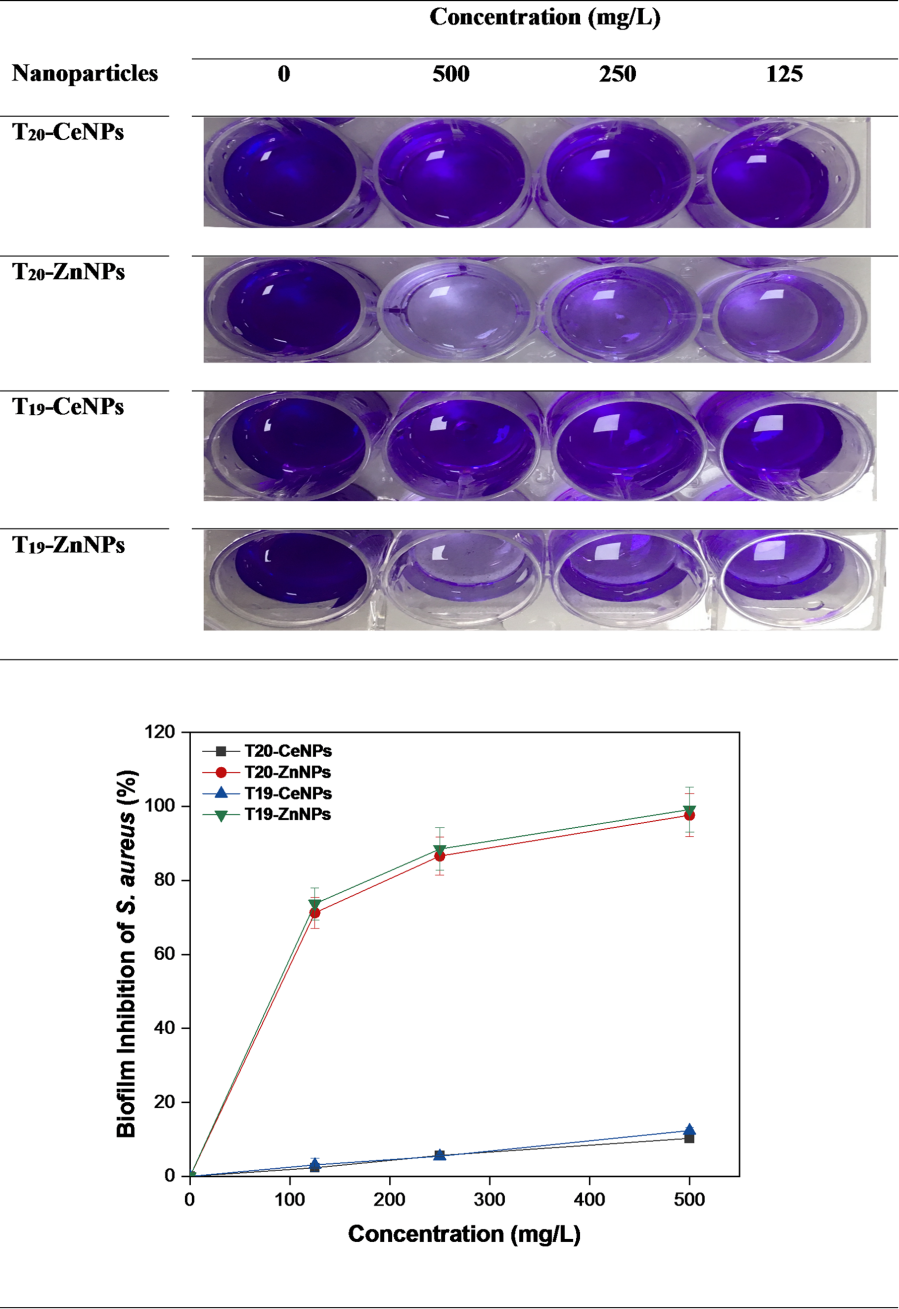
*S. aureus* biofilm inhibition of T_20_-CeNPs, T_20_-ZnNPs, T_19-_CeNPs, and T_19_-ZnNPs

Biofilm is defined as the microorganism that adheres to a surface and the collection of materials that they produce (Heydorn et al. 2000). It is widely accepted that genes regulating the production of extracellular polymeric substances (EPS) influence biofilm formation (Branda et al. 2005). About 80% infection of bacteria occurs by biofilms. Therefore, the development of biofilms has clinical significance (Schachter 2003). So, the impact of T_19_-CeNPs, T_19_-ZnNPs, T_20_-CeNPs, and T_20_-ZnNPs on biofilm inhibition against *P. aeruginosa* and *S. aureus* was studied at different concentrations. The result of biofilm inhibition of *P. aeruginosa* and *S. aureus* is shown in Figs. 9 and 10. As seen in Fig. 9, the biofilm inhibition of T_19_-CeNPs and T_19_-ZnNPs were found as 18.67% and 60.53% at 125 mg/L, 38.68% and 80.39% at 250 mg/L and 53.2% and 95.67% at 500 mg/L for *P. aeruginosa*, respectively and the effect of T_20_-CeNPs and T_20_-ZnNPs on biofilm inhibition of *P. aeruginosa* were 6.41% and 45.73% at 125 mg/L, 12.39% and 69.37% at 250 mg/L, and 21.38% and 78.49% at 500 mg/L, respectively. It was also investigated to affect of T_19_-CeNPs, T_19_-ZnNPs, T_20_-CeNPs, and T_20_-ZnNPs on biofilm inhibition of *S. aureus* and T_19_-ZnNPs and T_20_-ZnNPs displayed effective biofilm inhibition activity as 73.68% and 71.23% at 125 mg/L and 88.49% and 86.59% at 250 mg/L, respectively. It was found that the highest biofilm inhibition was obtained with T_19_-ZnNPs and T_20_-ZnNPs as 99.14% and 97.64% at 500 mg/L against *S. aureus*, respectively. ZnNPs showed higher biofilm inhibition than CeNPs against both tested bacteria. Obeizi et al. (2020) obtained essential oil extract from *Eucalyptus globulus* leaves by hydrodistillation and they synthesized ZnONP by green synthesis method using essential oil extracts. They also investigated the biofilm inhibition of green synthesized ZnONP and it was found that the biofilm inhibition value of ZnONP obtained from *Eucalyptus globulus* was 85% for *S. aureus* and 97% for *P. aeruginosa*. Altaf et al. (2021) synthesized CeONPs by using green process from *Acorus calamus* extract and they characterized them with UV–visible spectroscopy, X-ray diffraction, FTIR, and TEM analyzes. They studied the effect of green synthesized CeONPs on biofilm inbition of several patogen microorganisms. They found the biofilm inhibition value of CeONPs synthesized from *Acorus calamus* to be 30.07% for *P. aeruginosa* and 37.31% for *S. aureus* at 400 µg/mL. The results of this investigations showed that a significant antibiofilm ability was obtained with green synthesized of ZnONPs from thermophilic *A. flavithermus* strain Gecek19 and *G. stearothermophilus* strain Gecek 20 both against Gram positive ve and Gram negative bacteria. In conclusion, T_19_-ZnNPs, T_20_-CeNPs, and T_20_-ZnNPs can be used for biofilm inhibition in several industries such as environmental and healthy.

### Microbial cell viability inhibition

**Fig. 11:**
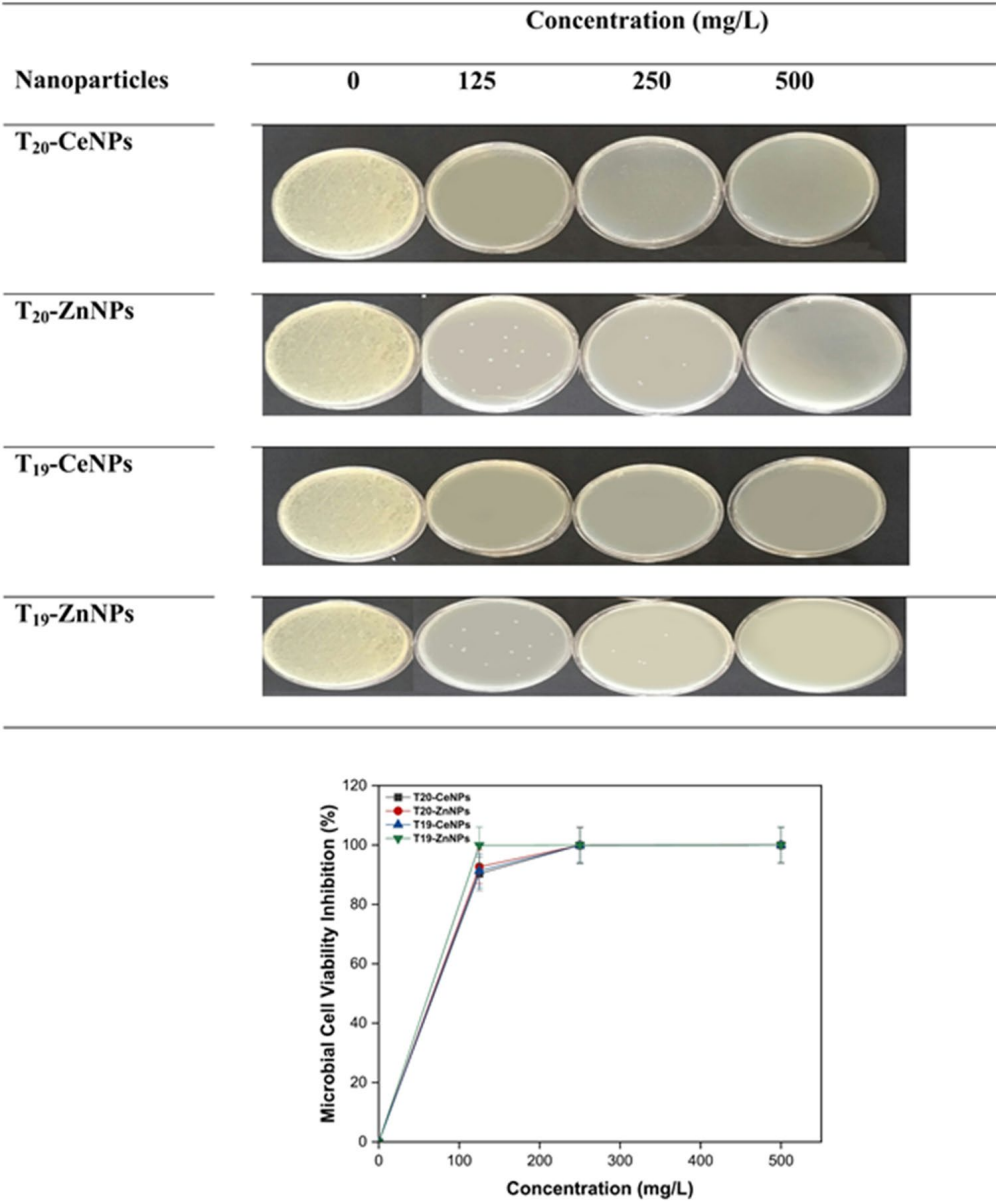
Microbial cell viability inhibition of T_20_-CeNPs, T_20_-ZnNPs, T_19-_CeNPs, and T_19_ ZnNPs

In this study, the effects of T_19_-CeNPs, T_19_-ZnNPs, T_20_-CeNPs, and T_20_-ZnNPs on the viability inhibition of *E. coli* were investigated at different concentrations. Microbial cell viability inhibition is shown in Fig. 11. It was found that T_19_-CeNPs, T_19_-ZnNPs, T_20_-CeNPs, and T_20_-ZnNPs inhibited microbial growth as 91.34%, 99.91%, 90.35%, and 92.67% at 125 mg/L, respectively and also *E. coli* was inhibited as 100, 100, 99.99, and 100% at 500 mg/L by T_19_-ZnNPs, T_20_-CeNPs, and T_20_-ZnNPs, respectively when compared with control. Navale et al. (2015) syhthesized ZnONP and they also investigated the effects ZnONP on the viability inhibition of *S. aureus* and *S. typhimurium* microbial cells. They incubated *S. aureus* with ZnONP at concentrations of 60 and 100 µg/mL for 4 h and *S. aureus* growth nearly inhibited as 50% and 95%, respectively when compared with control. In addition to these, they treated *S. typhimurium* with newly synthesized ZnNPs for 4 h at 80 and 100 mg/L. It was found that *S. typhimurium* viability decreased 75% in presence of 80 mg/L and 90% in presence of 100 mg/L when compared with control. Pelletier et al. (2010) informed that they synthesized and characterized CeONP. They studied the microbial cell viability of CeONP at different concentrations by using *E. coli* and *B. subtilis* as microbial strains. They stated that the microbial cell viability was concentration dependent and they observed microbial cell viability inhibition. Our results indicated that T_19_-ZnNPs, T_20_-CeNPs, and T_20_-ZnNPs can be used for inactivation of microbial cell for several purposes.

### Seed germination and root/coleoptile development

**Fig. 12:**
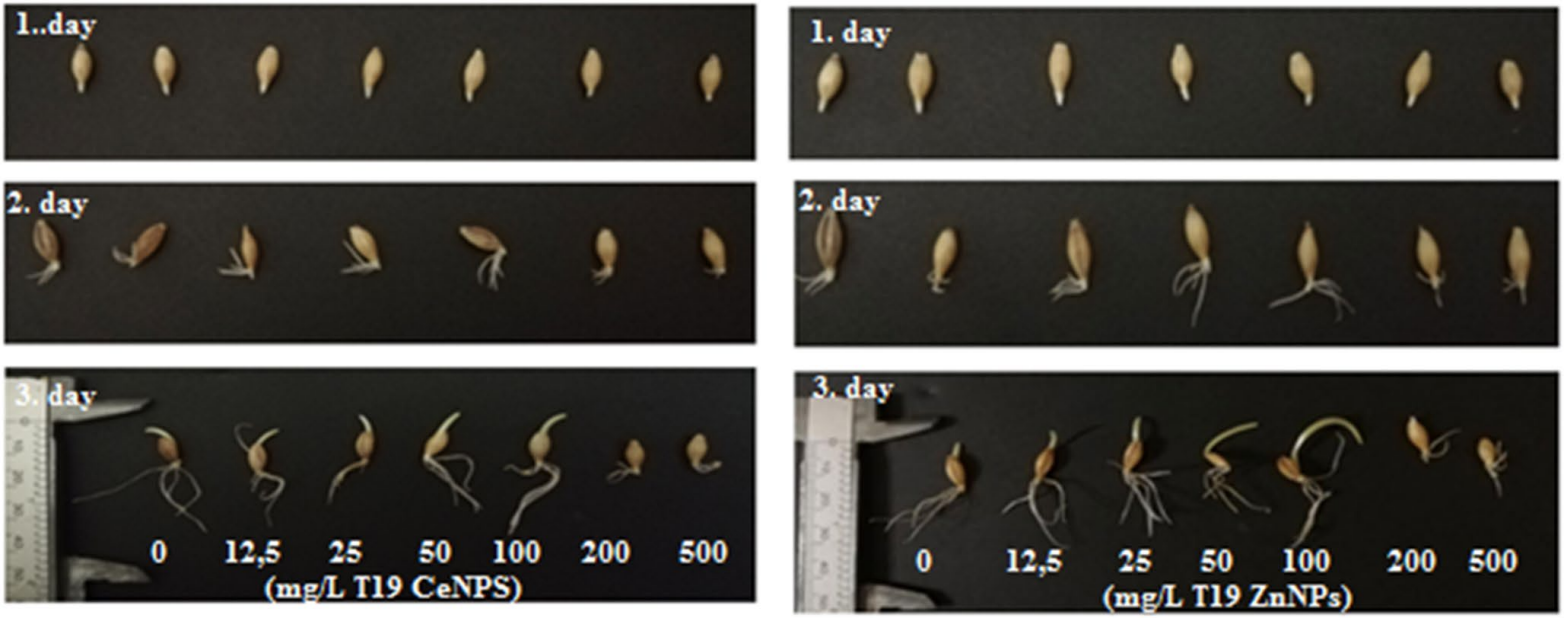
Phenotypic images of barley seedlings treated with different concentrations of T19NPs for 3 days

**Fig. 13:**
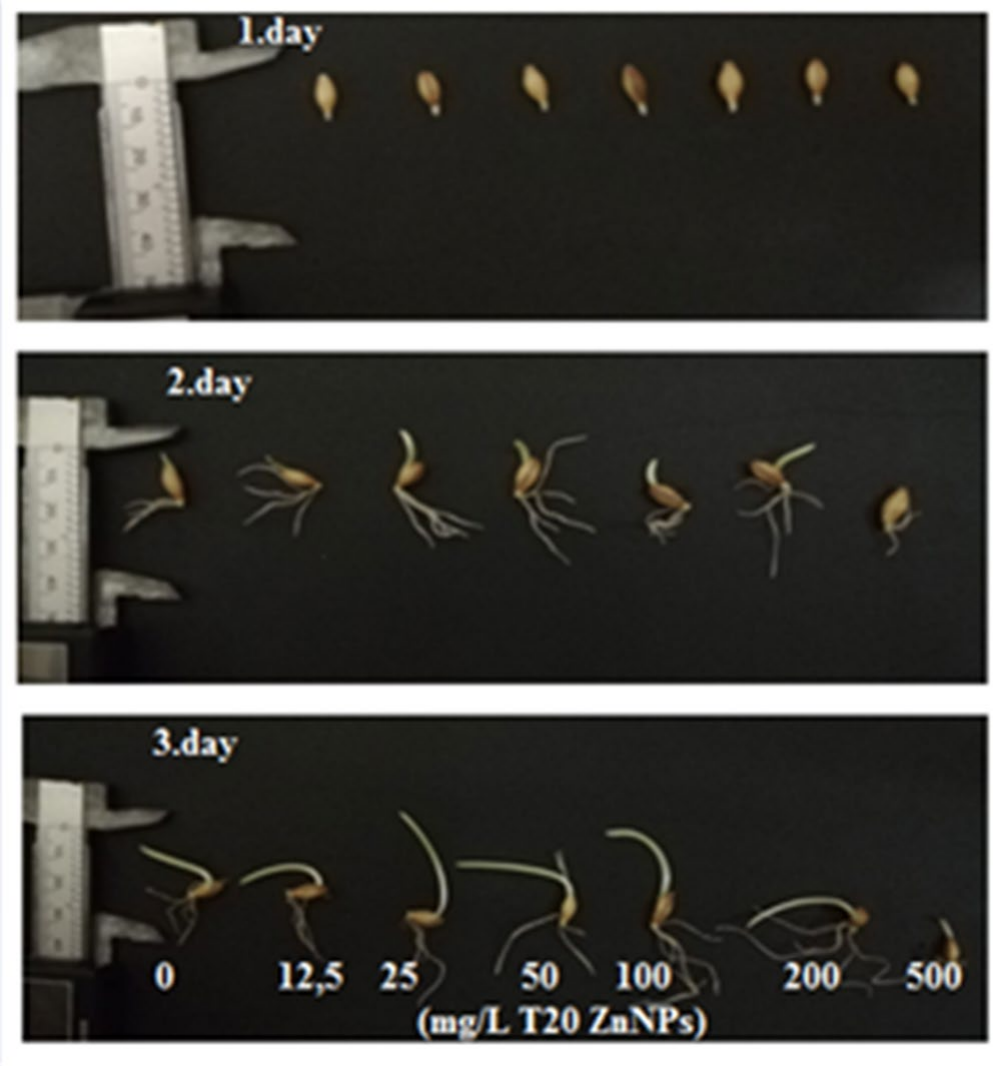
Phenotypic images of barley seedlings treated with different concentrations of T20NPs for 3 days

**Fig. 14:**
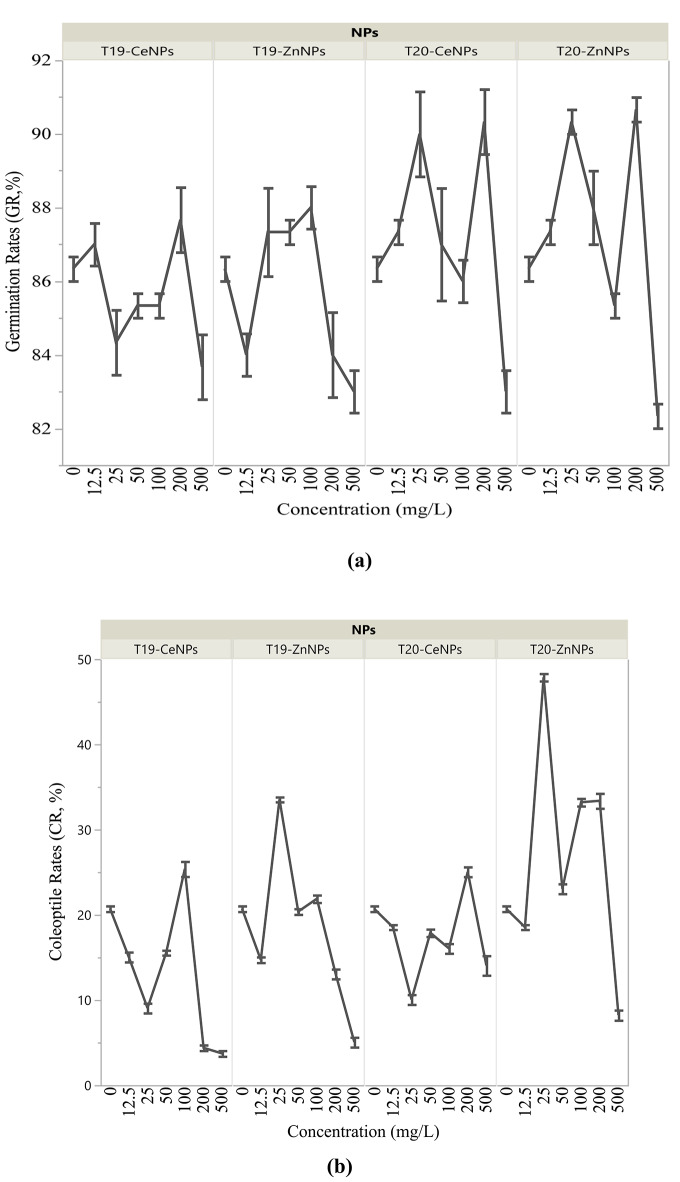

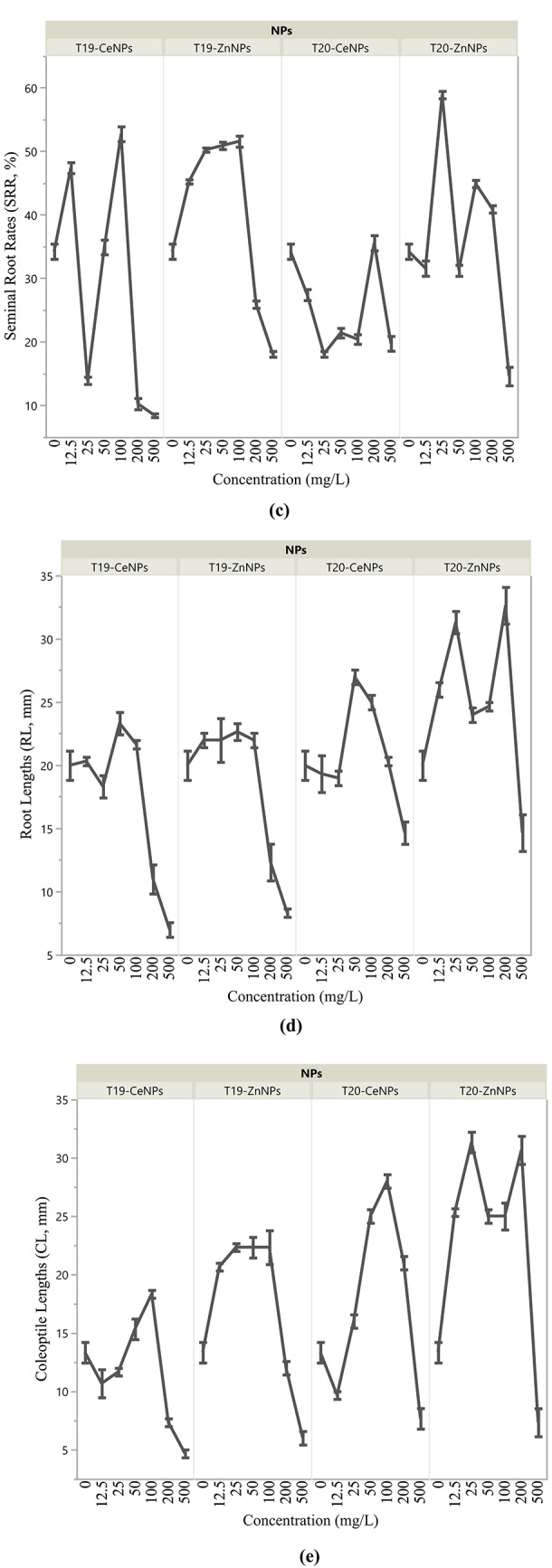
Effect of different concentration of T19 CeNPs, T19 ZnNPS, T20 CeNPS, T20 ZnNPs on barley seedlings’ **a** Germination Rates (GR, %), **b** Coleoptile Rates (CR, %) and **c** Seminal Root Rates (SRR, %), **d** Root Lengths (RL, mm) and **e** Coleoptile Lengths (CL, mm)

This study was carried out to evaluate the concentration-dependent effects of Ce and Zn nanoparticles obtained from two different bacterial strains at the initial stage of plant development. The impacts of all these nanoparticles on germination, rooting and coleoptile formation were photographed on a day basis (Figs. 12 and 13). The effects of Ce and Zn nanoparticles obtained from two different bacterial strains (T19, T20) with different concentration (12.5-25-50-100-200-500 mg/L) were found statistically significant in terms of germination and early seedling development in barley crops (Table 2). Germination rates were found to be between 82% and 91% (Fig. 14a). The highest germination rate was observed in nanoparticles obtained from T20 strain. The highest coleoptile rate was measured T20 ZnNPs applications at 25 mg/L concentration, with an increase of approximately two-fold compared to the control (Control = 21%, T20 NPs = 48%) (Fig. 14b). The highest seminal root rate was measured T20 ZnNPs applications at 25 mg/L concentration, with an increase of 1.7 fold compared to the control (Control = 34%, T20 NPs = 59%) (Fig. 13c). The highest root and coleoptile lenght were measured T20 ZnNPs applications at 25 mg/L and 200 mg/L concentration (Figs. 14d, e). In ZnNPS and CeNPs obtained from T19 bacterial strain, it was observed that the concentrations of 200 mg/L and 500 mg/L significantly reduced CR, SRR and RL, CL compared to the control.

**Table 2:** Effect of green synthesised CeNPs and ZnNPs at different concentrationon on the germination and growth parameters of barley seedlings

Bacterial strains	NPs	Concentration	Germination Rates (GR, %)	Coleoptile Rates (CR, %)	Seminal Root Rates (SRR, %)	Root Lengths (RL, mm)	Coleoptile Lengths (CL, mm)
T19	CeNPs	0	86 (68.3)b-e	21 (27.1)e	34 (35.9)fg	20.0 g-j	13.3gh
		12,5	87 (68.9)bcd	15 (22.8)gh	48 (43.6)cd	20.3 g-j	10.7ij
		25	84 (66.7)e-h	9 (17.4)jk	14 (22.0)l	18.3j	11.7hij
		50	85 (67.5)d-g	16 (23.2)gh	35 (36.3)f	23.3c-f	15.3 fg
		100	85 (67.5)d-g	25 (30.2)c	53 (46.6)b	21.7f-i	18.3e
		200	88 (69.5)bc	4 (12.0)lm	10 (18.7)m	11.0 lm	7.3 L
		500	84 (66.2)gh	4 (11.0)m	9 (17.0)n	7.0n	4.7 m
	ZnNPs	0	86 (68.3)b-e	21 (27.1)e	34 (35.9)fg	20.0.g-j	13.3gh
		12,5	84 (66.5)fgh	15 (22.5)gh	45 (42.3)d	22.0e-h	20.7d
		25	87 (69.2)bcd	34 (35.4)b	50 (45.2)bc	22.0e-h	22.3d
		50	87 (69.2)bcd	20 (26.8)e	51 (45.6)b	22.7d-g	22.3d
		100	88 (69.7)bc	22 (27.9)de	52 (46.0)b	22.0e-h	22.3d
		200	84 (66.5)fgh	13 (21.1)i	26 (30.7)i	12.3kl	12.0hi
		500	83(65.7)h	5 (12.8)l	18 (25.2)k	8.3mn	6.0 lm
T20	CeNPs	0	86 (68.3)b-e	21 (27.1)e	34 (35.9)fg	20.0 g-j	13.3gh
		12,5	87 (69.2)bcd	19 (25.5)f	28 (31.6)i	19.3hij	9.7jk
		25	90 (71.7)a	10 (18.4)j	18 (25.2)k	19.0ij	16.0f
		50	87 (69.0)bcd	18 (25.0)f	22 (27.6)j	27.0b	25.0c
		100	86 (68.1)c-f	16 (23.6)g	21 (26.9)j	25.0bcd	28.0b
		200	90 (71.9)a	25 (30.0)c	36 (37)f	20.3 g-j	21.0d
		500	83 (65.7)h	14 (21.9)hi	20 (26.4)jk	14.6k	7.6kl
	ZnNPs	0	86 (68.3)b-e	21 (27.1)e	34 (35.9)fg	20.0 g-j	13.3gh
		12,5	87 (69.2)bcd	19 (25.5)f	32 (34.2)gh	26.0bc	25.3c
		25	90 (71.9)a	48 (43.8)a	59 (50.2)a	31.3a	31.3a
		50	88 (69.8)b	23 (28.7)d	31 (34.1)h	24.0c-f	25.0c
		100	85 (67.5)d-g	33 (35.2)b	45 (42.2)d	24.7b-e	25.0c
		200	91 (72.2)a	33 (35.3)b	41 (39.8)e	32.7a	30.7a
		500	82 (65.2)h	8 (16.6)k	15 (22.5)l	14.6k	7.3 L
LSD			1.711**	1.253***	1.674***	2.671***	2.186***

Zinc (Zn) is a necessary microelement for plant growth and very important for food crops sustainability (Sharma et al. 2013). Itroutwar et al. (2020), in their study with biogenic ZnO NPs at different concentrations (10, 25, 50, 100, and 200 mg/L), found that 100 mg/L NPS application showed nanopriming effect and improved germination percentage, root and shoot length. Singh et al. (2019) reported this value as 62.5 mg/L for ZnO NPs. Asmat-Campos et al. (2022), they reported this value as about 100 mg/L for ZnO NPS. García-López et al. (2019) reported this value for ZnO NPs between 0 and 300 mg/L and described 400–500 mg/L as toxic. Concentration ranges given in other studies are consistent with this study. With the widespread in the use of nano cerium in different fields, environmental effects and agri-food indusrty usage potentials of these nanoparticles have been the subject of research and curiosity. In this context, the most basic studies that can be done with plants are germination and early growth studies. In the studies with cerium, it has been revealed by different researchers that it enhances seed germination, development of seedling roots and growth indexes (Yang et al. 2020; Ramírez-Olvera et al. 2018; Lizzi et al. 2018). Yang et al. (2020) reported that as a result of CeO_2_ nanoparticle applications, the highest growth index was measured at 100 mg/L concentration and the lowest growth index was measured at 500 mg/L concentration. In this study, it was observed that CeNPS applications at 50 and 100 mg/L concentrations increased rooting, which supports other research results.

In this study, when a general evaluation is made, it can be said that the application of ZnNPS as type of nanoparticle is more advantageous in terms of supporting the plant than the application of CeNPs. When compared on the basis of bacterial strain, it can be said that T20 strain stands out more than T19 strain. It was observed that all data were negatively affected and suppressed at the highest concentration of 500 mg/L. In this study, 500 mg/L concentration was determined as inhibitory dose for all administration groups. It is seen that when used in effective concentrations (between 25 and 100 mg/L in both NPS), it can increase rooting and leaf formation in the cereals. In the different studies have reported different effects of ZnNPS and CeNPS on crops, especially according to the concentration, size and type of the nanoparticles (Lizzi et al. 2018; Prasad et al. 2012; Yang et al. 2020; Ramírez-Olvera et al. 2018). Due to the characteristic aggregation properties of nanoparticles and their different effects in each organism, it is not easy to study and therefore many studies are required to do, this study is one of them. The present results suggest that nano Zn and nano Ce shows a concentration-dependent stimulating effect during the germination and early seedling growth period.

### Application potential and economic viability

The practical applicability of biosynthesized ZnNPs and CeNPs depends not only on their biological performance but also on their economic feasibility and scalability. In this context, a comparative cost perspective between conventional chemical synthesis and the present extracellular biosynthesis route is essential. Conventional ZnO nanoparticle synthesis methods (e.g., sol–gel, hydrothermal, and precipitation) typically require high-purity chemical reagents (e.g., zinc acetate), organic solvents, and additional stabilizing agents (Swain et al. 2025). Reported production costs in laboratory-to-pilot scale systems are generally in the range of ~ 50–150 USD per kg of ZnO nanoparticles, depending on purity, energy input, and post-processing requirements. A significant fraction of this cost arises from chemical reagents (30–40%) and energy-intensive steps such as calcination (> 400 °C), which can account for an additional 20–30% of total production costs (Zahra et al. 2020).

In contrast, the extracellular green synthesis approach used in this study reduces both chemical and energy inputs. The process operates at moderate temperatures (55–70 °C) and eliminates the need for external reducing and capping agents. The primary cost components in this system are the metal precursor and microbial cultivation. For example, zinc sulfate (ZnSO₄) is a low-cost precursor (typically ~ 1–3 USD per kg at bulk scale), and nutrient broth-based microbial cultivation can be estimated at ~ 5–15 USD per kg of produced nanoparticles when scaled. Based on these considerations, the projected production cost of biosynthesized ZnO nanoparticles can be reasonably estimated in the range of ~ 15–40 USD per kg, representing a potential cost reduction of approximately 40–70% compared to conventional methods. Beyond direct production cost, additional economic advantages arise from process simplification and environmental compatibility (Ekins and Zenghelis, 2021). The absence of toxic chemicals reduces waste treatment costs, while the use of thermophilic bacteria enables operation under conditions that minimize contamination risk, thereby lowering sterilization and operational expenses.

From an application perspective, the synthesized ZnO nanoparticles demonstrated strong antimicrobial, antibiofilm (up to 99%), and plant growth-promoting effects, indicating high potential for use in agricultural nanofertilizers, seed priming agents, and antimicrobial coatings. In agriculture, even low-dose applications (25–100 mg/L) were sufficient to enhance germination and seedling development, suggesting that relatively small quantities of material are required per unit area, further improving cost-effectiveness at field scale.

Moreover, the high antibiofilm activity supports potential applications in medical coatings, water treatment systems, and food packaging, where prevention of microbial colonization is critical. In such applications, the durability and multi-mechanistic antimicrobial action of ZnO nanoparticles may reduce the need for repeated chemical treatments, providing additional indirect economic benefits.

Despite these advantages, certain challenges remain for full industrial implementation. These include scale-up of bioreactor systems, consistency in nanoparticle size distribution, and variability in extracellular metabolite composition. Therefore, further techno-economic analysis and life cycle assessment studies at pilot scale are recommended to validate the long-term economic competitiveness of this approach.

Overall, the results of this study suggest that thermophilic bacteria-mediated nanoparticle synthesis represents a promising, cost-effective, and environmentally sustainable alternative to conventional production methods, with significant potential for applications in agriculture, environmental management, and antimicrobial technologies.

## Conclusion

Since there are no sufficient studies in the literature with nanoparticle synthesis using thermophilic microorganisms, this study is quite original. Different biological activities of T_19_-CeNPs, T_19_-ZnNPs, T_20_-CeNPs, and T_20_-ZnNPs such as antioxidant, DNA cleavage, antimicrobial, microbial cell viability and biofilm inhibition activities were tested at various concentrations. T19 and T20 are different microorganisms and synthesized different extracellular metabolic products during the 48-hour incubation period. It is not expected that CeNPs and ZnNPs will have the same biological activity using these extracellular fluids synthesized via the green synthesis method. Because these two nanoparticles have different chemical natures, redox properties, and cellular interaction mechanisms. The observed differences in biological activities between cerium oxide nanoparticles (CeNPs) and zinc oxide nanoparticles (ZnNPs) can be attributed to their distinct chemical natures, redox properties, and cellular interaction mechanisms. These differences directly influence their interactions with biomolecules, cellular uptake, and mechanisms of action. Furthermore, particle size, surface charge, and morphology also play important roles. Smaller nanoparticles with higher surface area-to-volume ratios tend to exhibit increased biological activity due to enhanced cellular uptake and surface reactivity. Differences in zeta potential influence nanoparticle–cell membrane interactions, affecting internalization efficiency and intracellular localization. The obtained data suggest that when Zn and Ce nanoparticles are used in right/effective concentrations, they can be used to support plant development in the germination and growth of cereal such as barley. According to these results, especially ZnNPs synthesized from thermophilic microorganisms may be candidates for various purposes in the agriculture and nanodrug industry.

## Data Availability

The datasets generated during and/or analyzed during the current study are available from the corresponding author on reasonable request.
